# Effect of light walking combined with arthroscopic surgery on bone mineral density in patients with foot and ankle osteoarthritis

**DOI:** 10.1016/j.heliyon.2024.e35575

**Published:** 2024-08-02

**Authors:** Xiaoyan Li, Kangwon You

**Affiliations:** a Department of Physical Education, Jinzhong University, Jinzhong 030619, China; b Department of Physical Education, Jeonju University, Jeonju 55069, South Korea

**Keywords:** Mild walking, Arthroscopic surgery, Foot and ankle osteoarthritis, BMD

## Abstract

ObjectiveTo observe the effect of light walking combined with arthroscopic surgery on the efficacy and Bone Mineral Density (BMD) of patients with ankle arthritis. **Methods** 180 patients with ankle arthritis were retrospectively selected, who were divided into a control group (Group A) and an experimental group (Group B) according to treatment methods, with 90 patients in each group. Group A was treated with conventional open surgery and Group B was treated with light walking combined with arthroscopic surgery. The two groups were compared before and after treatment on scales such as the AOFAS ankle hindfoot score. Meanwhile, the patients' BMD and levels of inflammatory factors such as tumor cell necrosis factor-α (TNF-α) were compared before and after treatment. **Results** The Kofoed and AOFAS scale scores were higher for mild walking combined with arthroscopic surgery than for conventional treatment (*P* < 0.05), and their pain visual analogue VAS scores were lower than for conventional treatment (*P* < 0.05). In Group B, the postoperative BMD increase was significantly higher (*P* < 0.05). The R-value between PDGF bone growth factor and BMD was 0.957 and the R-value between VEGF growth factor and BMD was 0.903. The R-value between patient age and BMD was −0.936 and the R-value between patient BMI and BMD was −0.913. The treatment efficiency in Group B was 96.7 %. **Conclusion** The results prove that light walking combined with arthroscopic surgery is suitable for the surgical treatment of patients with ankle arthritis because it has a better therapeutic effect and makes patients' BMD level improved.

## 1 Introduction

Arthroscopic surgery has many advantages, such as less trauma, less bleeding and fewer complications for the patient, which facilitates recovery [1]. Therefore, arthroscopic surgery becomes the main treatment option for patients with ankle arthritis [2]. The use of arthroscopy facilitates the removal and repositioning of the free body during surgery [3]. Arthroscopy can also provide additional information to the health care provider in the diagnosis and treatment of non-fracture foot and ankle patients. The arthroscope can be used to obtain soft tissue damage during surgery and to reach areas that are difficult to reposition by incision to remove free joint debris and inflamed synovial membranes in the foot and ankle [4]. Postoperative rehabilitation is required after surgery for ankle arthritis. In post-operative rehabilitation, patients can be facilitated with light walking exercises to promote post-operative rehabilitation of patients with ankle arthritis, choosing a gradual post-operative rehabilitation from weak to strong exercise intensity. BMD can be used to assess bone mineral content and is a quantitative indicator to determine symptoms such as osteoporosis in the human body. The bone structure of joint will be damaged when a patient suffers trauma. The tissues of joint also begin to degenerate when blood clot or hematoma occurs at the fracture site, resulting in a decrease in BMD of joint. This decrease in BMD is a major cause of osteoporosis and other diseases, which has a more serious impact on older patients [5]. In elderly patients with foot and ankle fractures, BMD changes after surgical treatment, and the recovery of the joint can be evaluated quantitatively [6]. Ankle arthritis is mostly caused by trauma and chronic strain. The main symptoms are swelling and pain in the ankle joint for young patients, which worsens during movement. Patients generally experience redness, swelling, and pain, ranging from joint deformities to joint disabilities, seriously affecting their normal life. A strong correlation existed between the longevity of surgically implanted prostheses and the patient's BMD [7]. Meanwhile, a strong association existed between the ankle joint and BMD in adult men during running. Strengthening the ankle muscles during exercise and improving the function of the foot and ankle joint can prevent sports injuries. Therefore, it also needs to focus on the observation and analysis of BMD in patients with ankle arthritis after surgery in light walking training after arthroscopic surgery.

The purpose of this article is to observe the therapeutic effect of mild walking in combination with arthroscopic surgery on patients with ankle osteoarthritis and the impact on BMD. This can improve the treatment outcomes of patients and also increase their BMD level, making it suitable for surgical treatment of patients with ankle arthritis. The significance of this article is to provide more convenient and effective treatment methods for patients with ankle arthritis.

## 2 Data and methods

### 2.1 General information

180 patients with ankle arthritis treated from January in 2020 to January in 2022 were selected in the hospital. These patients with ankle arthritis were divided into Group A and Group B of 90 patients each according to the treatment methods. Group A was treated with conventional open surgery and Group B was treated with light walking combined with arthroscopic surgery. In Group A, there were 43 males and 47 females, (48.6 ± 11.5) years, 49 patients with left ankle arthritis and 41 patients with right ankle arthritis. Meanwhile, the average disease duration was (5.3 ± 4.6) months. In Group B, there were 42 males and 48 females, (47.9 ± 10.2) years, 47 patients with left ankle arthritis and 43 patients with right ankle arthritis. Meanwhile, the average disease duration was (5.1 ± 5.0) months. The general data had no statistical difference (*P* > 0.05). This study was approved by the Medical Ethics Committee. All patients signed informed consent forms.

**Fig. 1:**
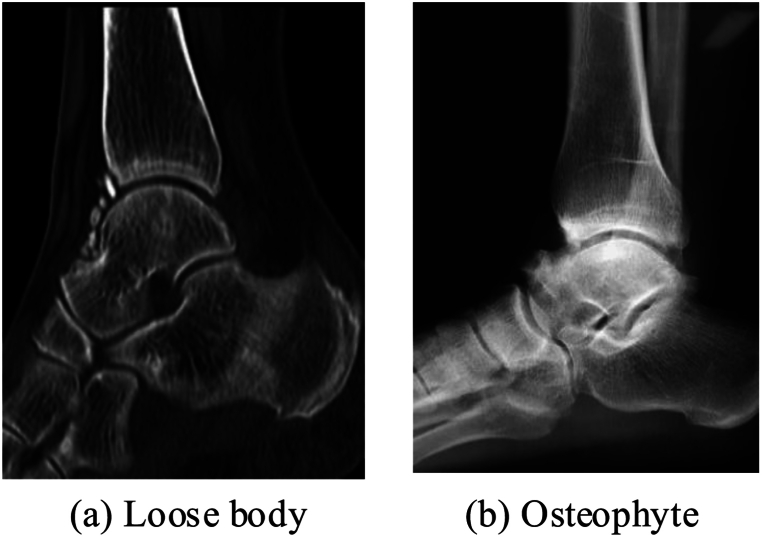
Main types of ankle arthritis in patients.

Inclusion criteria: (1) The patients exhibited symptoms of ankle pain, while the patients' ankle pain worsened after exercise and walking; (2) The patients had no history of ankle surgery, and all required arthroscopic surgery at the time of admission; (3) The patients' physical examinations all showed joint swelling, extensive pressure pain in joint space, and a sensation of friction with increased pain during activity; (4) The patients' physical examination findings were good, having no medical comorbidities; (5) After X-ray examination, the patient belonged to Takakura stage I of ankle arthritis. The main manifestation was parallel ankle joint, mainly formed by subchondral osteosclerosis or osteophytes. Exclusion criteria: (1) Patients with abnormal distal tibial force lines and requiring osteotomy; (2) Patients with co-infections such as soft tissue or joints; (3) Patients who were physically unable to undergo arthroscopic surgery; (4) Patients who also had ischemic diseases of the lower extremities; (5) Patients with coronary heart disease, diabetes mellitus, and other medical conditions. In Fig. 1, the main types of ankle arthritis that led to the patients in this study are shown, which are mainly caused by free bodies and bone fragments. Fig. 1(a) shows the loose body and Fig. 1(b) shows the osteophyte.

### 2.2 Research methodology

Group An underwent open surgery. Group B underwent surgery under arthroscopy and combined with light walking for rehabilitation. (1) Group A received conventional surgical treatment and underwent joint surface resection. (2) Group B received arthroscopic treatment. The patient remained in a supine position and underwent general anesthesia. Arthroscopic traction and fixation were performed at the lower thigh position, followed by ankle joint traction. Take the outer side as the entry point, then perform puncture and lumbar puncture needle insertion, and inject 20 mL of physiological saline into the joint space. With the help of vascular forceps, the soft tissue and joint capsule were completely separated. The arthroscope was inserted under direct vision after completion, with a depth of approximately 2.7 mm. Comprehensively observe the condition of the joint cavity and complete the planing of soft tissue with the help of arthroscopy. The surgeries mentioned in the article mainly involve arthroscopic debridement, without involving the spur removal or microfracture of the eccentric cartilage lesion. The content of arthroscopic debridement includes systematic examination of the joint cavity, removal of all cartilage debris and free bodies, removal of proliferative and edematous synovium, debridement of torn meniscus and removal of osteophytes causing mechanical disorders within the joint, and drilling treatment for subchondral bone exposure with a diameter of less than 1 cm. The patient underwent mild walking rehabilitation training after the surgery was completed.

Group B: (1) Basic care: The medical and nursing staff controlled the air, temperature, and humidity of the patient's ward to establish a relaxed therapeutic atmosphere, while guiding the patient's diet and strengthening the guidance. (2) Psychological intervention: Medical and nursing personnel communicated actively with patients and their conditions after surgery. Targeted psychological interventions were made to promote the expression of the patient's thoughts according to the actual physical condition and psychological state of the patient. Meanwhile, it was combined with listening to music, reading books, and other ways to divert the patient's attention to relieve the patient's psychological pressure. (3) Postoperative mild walking rehabilitation training: Encourage patients to carry out active postoperative recovery, select the intensity of exercise from weak to strong, strengthen functional exercise and enhance patients' self-confidence.

### 2.3 Observation indexes and judgment criteria

#### 2.3.1 Bone mineral density measurement

The patient's foot and ankle bone densitometry instrument was a dual-energy x-ray bone densitometer from Hologic, model Discover-Wi, USA.

#### 2.3.2 VAS score

The VAS score is often used in clinical applications to assess the level of pain in patients. The VAS score is simple to implement and has a high sensitivity of the assessment results. The initial end of the line is scored as 0 and the end of the line is scored as 10. The score of 0 indicates that patient feels no pain, 5 indicates that patient feels moderate pain, and 10 indicates that patient feels unbearable pain. Patients were asked to draw marks by their pain perception and the researcher scored the patient's pain level according to the marks. For example, if a patient marks at 1 cm, it means that the patient has a VAS score of 1.

#### 2.3.3 Kofoed score

The Kofoed scale has a high reliability and sensitivity and is commonly used in clinical practice for the assessment of osteoarthritis. The Kofoed scale includes three dimensions: patient's pain, joint function, and mobility. The Kofoed scale scores range from 0 to 100, with lower scores indicating lower bone and joint function, more severe pain, and lower mobility. Table 1 shows the specific scores of the Kofoed scale.

**Table 1:** Kofoed ankle function rating scale.

Item	Classification (score)	
Pain (single choice)	No pain (50 points)	
	Pain at the beginning of walking (40 points)	
	Pain when walking (35 points)	
	Occasionally pain occurs when walking with weight (35 points)	
	Pain every time when walking with weight (15 points)	
	Pain or persistent pain during examination (0 point)	
Function (multiple choice)	Walk with toes (3 points)	
	Walking with heel (3 points)	
	Go up and down stairs with normal gait (6 points)	
	Stand on one leg (6 points)	
	Walk without assistance (6 points)	
	Do not use orthopedic foot brace (6 points)	
Activity (multiple choice)	Ankle joint extension	Less than 10° (5 points) 5–9° (3 points) more than 50° (1 points)
	Ankle flexion	Less than 30° (5 points) 15–29° (3 points) more than 15° (1 points)
	Ankle rotation backward	Less than 30° (3 points) 15–29° (2 points) more than 15° (1 points)
	Pronation of ankle joint	Less than 20° (3 points) 10–19° (2 points) more than 10° (1 points)
	Turn out when carrying weight	More than 5° (2 points) 5–10° (1 points) less than 10° (0 points)
	Inversion when loading	More than 3° (2 points) 4–7° (1 points) less than 7° (0 points)

Note: Treatment effect evaluation criteria.

(1) Recovery: Kofoed score is 85–100 points (ankle pain, tenderness, congestion and swelling completely disappear, and move freely).

(2) Remarkable effect: Kofoed score is 75–84 points (ankle pain, tenderness, congestion and swelling basically disappear, and movement is slightly limited).

(3) Effective: Kofoed score is 70–74 points (ankle pain, tenderness, congestion and swelling are reduced, and activity is improved).

(4) Invalid: Kofoed score <70 (no remission of symptoms after treatment, and activity is still limited).

#### 2.3.4 AOFAS ankle hindfoot Score

The AOFAS scale was used in the study to evaluate the patient's ankle function. The recovery of the patient's ankle and hindfoot joints was assessed using this scale. The AOFAS scale was scored out of 100 points, with 50 points for ankle function, 40 points for the patient's pain, and 10 points for the patient's foot alignment. A score in the range of 90–100 indicates that the patient's ankle function is excellent, a score in the range of 75–89 indicates that the patient's ankle function is good, a score in the range of 50–74 indicates that the patient's ankle function is fair, and a score below 50 indicates that the patient's ankle function is poor. Table 2 shows the specific scores of the AOFAS scale.

**Table 2:** AOFAS ankle function rating scale.

Item	Classification	Score
Pain (40 points)	Nothing	40
	Mild, occasionally	30
	Moderate, common	20
	Severe, continuous	0
Function, autonomous activities and support (10 points)	No restriction, no support required	10
	Daily activities are not limited, entertainment activities are limited, and walking stick is required	7
	Daily activities are limited in weight production, and need to lift the car, assist the penalty, wheelchair, and support	0
Maximum walking distance (block) (5 points)	>6	5
	4∼6	4
	1∼3	2
	<1	0
Ground walking (5 points)	No difficulty	5
	When walking on uneven ground, stairs, quilts and ladders, people feel difficult.	3
	When walking on uneven ground, stairs, quilts and ladders, people feel very difficult.	0
Abnormal gait (5 points)	None, slight	8
	Obvious	4
	Remarkable	0
Forward and backward movement (flexion/extension) (8 points)	Normal or being restricted slightly (≥30°)	8
	Being limited moderately (15°–29°)	4
	Being restricted severely (<15°)	0
Rear foot movement (varus plus valgus) (6 points)	Normal or being restricted slightly (75 %∼100 % normal)	6
	Being limited moderately (25 %–74 % normal)	3
	Being restricted severely (<25 %)	0
Ankle-back foot stability (anteroposterior, varus - valgus) (8 points)	Stable	8
	Obvious instability	0
Foot alignment (10 points)	Excellent: metatarsal flexion, normal ankle-foot alignment	10
	Good: plantar flexion, ankle-posterior obvious angulation, asymptomatic	5
	Poor: non-plantar flexion, severe ankle-posterior alignment, symptomatic	3

Scores were measured on the Kofoed, AOFAS, and VAS scales before and after treatment for patients. The postoperative scale scoring time was 6 months and 12 months. The Kofoed and AOFAS scales were selected to evaluate the recovery of ankle joints in patients. At present, Kofoed and AOFAS scales are commonly used evaluation criteria, which can comprehensively reflect the patient's ankle joint recovery situation [8]. Therefore, the Kofoed and AOFAS scales were used for evaluation in this experiment. Due to the subjectivity of the AOFAS scale, it was used as an auxiliary evaluation result in the experiment. The experimental results were evaluated by combining indicators such as VAS and inflammatory factors, ultimately determining the patient's recovery status comprehensively.

#### 2.3.5 Biochemical index test

Inflammatory markers, such as hs-CRP, TNF-α, and IL-6, as well as growth factors, such as PDGF and VEGF were measured using Enzyme Linked Immunosorbent Assay (ELISA) method. Both groups of patients were drawn 1–2 ml of blood before and after treatment. The testing time was before surgery, 1 month after surgery, and 2 months after surgery. ELISA was used for detection. Please refer to the instruction manual for specific testing methods. The levels of cytokines such as IL-6, TNF-a, PDGF, and VEGF in the knee joint fluid of two groups of patients were measured.

### 2.4 Statistical analysis

SPSS 22.0 software was used for data processing. When the patient's measurement data conformed to a normal distribution, $\bar{\chi }\pm s$ was used to represent these data, and the inter group comparison was completed through *t*-test. When the patient's measurement data did not follow a normal distribution, they were represented by the median. The component comparison was completed through Wilcoxon rank sum test. The counting data were expressed as a percentage and inter group comparison was completed through chi square test. The test level of the data was α = 0.05, and *P* < 0.05 indicated that the measured experimental results were statistically significant.

The experimental results were evaluated by two experts simultaneously. When the opinions of two experts were consistent, it could be considered as the final experimental result. When there was a disagreement between the two, a third party was selected for evaluation to reduce bias in the experimental results.

## 3 Results

### 3.1 Comparison of general information of patients in each group at the time of admission

180 patients were divided into two groups, each group having 90 patients. There were no statistically significant differences (*P* > 0.05) in terms of gender, age, mean disease duration, BMI, VAS rating scale, Kofoed rating scale, AOFAS rating scale, and general information including inflammatory factors such as TNF-α, IL-6, and PDGF and VEGF. They could be used in the follow-up comparative experiments, as detailed in Table 3.

**Table 3:** Comparison results of general data.

Information	Classification	Group A	Group B	χ^2^*/t*	*P*
Gender	Male	42	43	0.376	0.756
	Female	48	47		
Ankle	Left ankle	47	49	0.465	0.683
	Right ankle	43	41		
Age (year, $\bar{x}$ ±s)	–	47.9 ± 10.2	48.6 ± 11.5	0.879	0.059
Average course of disease (months, $\bar{x}$ ±s)	–	5.1 ± 5.0	5.3 ± 4.6	0.532	0.408
BMI (kg/m^2^)	–	22.86 ± 2.58	23.01 ± 2.01	1.072	0.053
VAS score	–	7.15 ± 2.33	7.29 ± 1.15	0.423	0.352
Kofoed score	–	23.97 ± 4.61	24.16 ± 5.17	0.618	0.567
AOFAS score	–	37.26 ± 6.38	37.82 ± 5.21	0.552	0.639
Biochemical index	hs-CRP (g/cm^2^)	12.68 ± 2.34	12.57 ± 2.56	0.465	0.314
	TNF-α (g/cm^2^)	14.92 ± 3.06	14.62 ± 2.66	0.503	0.298
	IL-6 (g/cm^2^)	23.58 ± 2.67	23.19 ± 2.99	0.482	0.301
	PDGF(U/L)	159.88 ± 23.19	157.67 ± 20.77	0.298	0.706
	VEGF (pg/mL)	55.32 ± 10.82	53.12 ± 11.03	0.194	0.873

### 3.2 Comparison of each rating scale between Group A and Group B

In Table 4, the results of each score scale were compared after the corresponding surgery and treatment. At 6 months after surgery, the VAS rating scale was lower in two groups than before surgery (*P* < 0.05). The Kofoed and the AOFAS rating scales were higher in both Groups A and B than before surgery (*P* < 0.05). At 12 months after surgery, the VAS rating scale was lower in the two groups (*P* < 0.05). The Kofoed rating scale and the AOFAS rating scale were higher in two groups (*P* < 0.05). During the same period, the Kofoed and AOFAS scale scores were higher in Group B than the Kofoed and AOFAS scale scores in Group A (*P* < 0.05). The VAS scale scores in Group B were lower than VAS scale scores in Group A (*P* < 0.05).

**Table 4:** Comparison of the scoring scales.

Index	Time	Group A	Group B	*t*	*P*
VAS score	Before operation	7.15 ± 2.33	7.29 ± 1.15	0.423	0.352
	6 months after operation	5.03 ± 1.27a	3.16 ± 0.87ac	6.498	0.001
	12 months after operation	3.01 ± 0.62 ab	1.98 ± 0.32 abc	6.618	0.001
Kofoed score	Before operation	23.97 ± 4.61	24.16 ± 5.17	0.618	0.567
	6 months after operation	52.69 ± 6.91a	65.88 ± 7.32ac	6.312	0.001
	12 months after operation	69.83 ± 4.54 ab	82.17 ± 8.65 abc	7.981	0.001
AOFAS score	Before operation	37.26 ± 6.38	37.82 ± 5.21	0.552	0.639
	6 months after operation	49.32 ± 5.34a	59.82 ± 7.94ac	9.129	0.001
	12 months after operation	67.58 ± 8.12 ab	80.33 ± 10.95 abc	8.423	0.001

Note: ^a^*P* < 0.05 compared with the same group before operation; ^b^*P* < 0.05 compared with the same group 6 months after operation; ^c^*P* < 0.05 compared with the control group of the same period.

### 3.3 Comparison of each biochemical index between two groups

Results of various biochemical indicators including TNF-α, IL-6 and other inflammatory factors and PDGF and VEGF in patients in different groups were compared in Table 5. After 1 month of surgical treatment, inflammatory factor levels decreased and growth factor levels increased in both groups compared to the pre-experimental period (*P* < 0.05). After 2 months after surgical treatment, inflammatory factor levels decreased and growth factor levels increased compared with the pre-experimental and 1 month post-surgical periods (*P* < 0.05). During the same period, inflammatory factors in Group B were all lower than the levels of inflammatory factors in Group A (*P < 0*.05), and growth factors in Group B were all higher than the levels of growth factors in Group A (*P* < 0.05).

**Table 5:** Comparison of biochemical indexes.

Index	Time	Group A	Group B	*t*	*P*
hs-CRP (g/cm^2^)	Before operation	12.68 ± 2.34	12.57 ± 2.56	0.465	0.314
	1 month after operation	8.53 ± 1.28a	6.98 ± 1.36ac	7.132	0.001
	2 months after operation	6.72 ± 1.28 ab	4.17 ± 0.78 abc	9.312	0.000
TNF-α (g/cm^2^)	Before operation	14.92 ± 3.06	14.62 ± 2.66	0.503	0.298
	1 month after operation	9.69 ± 1.15a	7.01 ± 1.34ac	6.329	0.001
	2 months after operation	7.06 ± 1.27 ab	5.12 ± 0.98 abc	8.187	0.000
IL-6 (g/cm^2^)	Before operation	23.58 ± 2.67	23.19 ± 2.99	0.482	0.301
	1 month after operation	16.42 ± 1.18a	13.25 ± 1.46ac	8.369	0.001
	2 months after operation	8.14 ± 0.97 ab	5.36 ± 1.01 abc	10.315	0.000
PDGF(U/L)	Before operation	159.88 ± 23.19	157.67 ± 20.77	0.298	0.706
	1 month after operation	183.24 ± 26.58a	198.52 ± 26.96ac	6.319	0.001
	2 months after operation	213.65 ± 21.78 ab	298.65 ± 21.39 abc	14.829	0.000
VEGF (pg/mL)	Before operation	55.32 ± 10.82	53.12 ± 11.03	0.194	0.873
	1 month after operation	100.82 ± 21.57a	132.61 ± 25.49ac	7.982	0.001
	2 months after operation	157.66 ± 28.56 ab	209.51 ± 27.83 abc	16.943	0.000

Note: ^a^*P* < 0.05 compared with the same group before operation; ^b^*P* < 0.05 compared with the same group 6 months after operation; ^c^*P* < 0.05 compared with the control group of the same period.

### 3.4 Comparison of bone mineral density between two groups

**Fig. 2:**
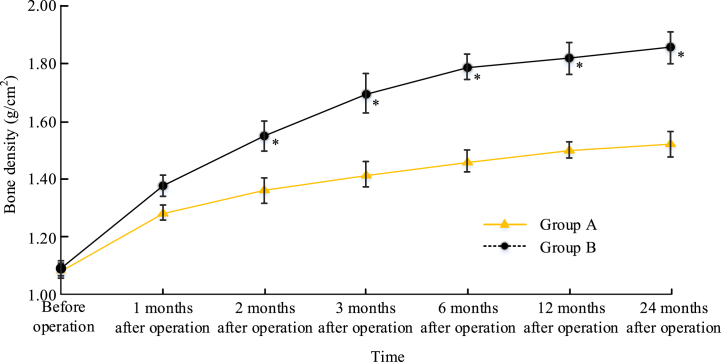
BMD of the two groups at different time periods Note: *P < 0.05.

The changes in BMD between the two groups at different time periods were compared in Fig. 2. There was no significant difference between the BMD of the two groups before the surgery. At 1 month after the surgical treatment, the BMD of Group B was higher, while *P* > 0.05. At 2 months after surgery, the BMD increase in Group B was significantly higher (*P* < 0.05). The trend of BMD growth was more pronounced in the first 6 months after surgery, and the rate of BMD growth stabilized in both Groups A and B after 6 months.

### 3.5 Correlation study of BMD and growth factors in patients

**Fig. 3:**
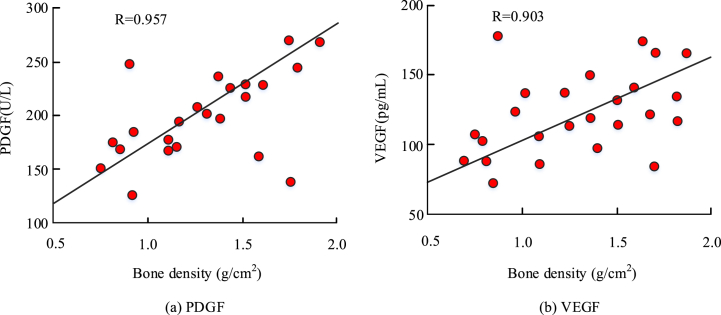
Correlation between bone mineral density and growth factors.

The results of the correlation analysis between PDGF bone growth factor, VEGF growth factor and BMD are shown in Fig. 3. Fig. 3(a) shows the results of the correlation analysis between PDGF bone growth factor and BMD. Fig. 3(b) shows the results of the correlation analysis between VEGF growth factor and BMD. The R-value between PDGF bone growth factor and BMD was 0.957, and the R-value between VEGF growth factor and BMD was 0.903, both of which had some positive correlation.

### 3.6 Correlation study between patients' BMD and age and BMI

**Fig. 4:**
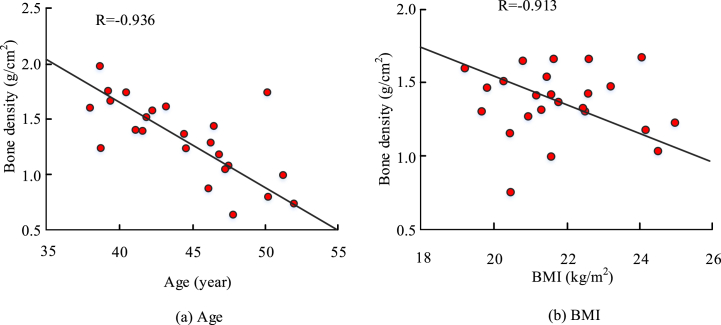
Results of correlation between BMD and age, BMI.

The results of the correlation analysis between the patient's age and BMD and the correlation analysis between the patient's BMI value and BMD were presented in Fig. 4. The R-value between the patient's age and BMD was −0.936, and the R-value between the patient's BMI value and BMD was −0.913, which showed some negative correlation.

### 3.7 Comparative results of patient efficacy and adverse effects

In Table 6, the incidence of adverse reactions and treatment of patients are presented. Dizziness and abnormal autonomic reflexes occurred in both groups, while no significant difference existed and incidence rate was low. The treatment efficiency of the two groups were above 90 %, and the treatment efficiency of Group B was 96.7 %, which was relatively high.

**Table 6:** The comparison results of the efficacy and adverse reactions.

Index	n	Classification	Group A	Group B	*χ2*	*P*
Adverse reaction rate	90	Dizzy	2	1	–	–
		Abnormal autonomic reflex	1	1	–	–
		Total incidence (n/%)	3 (3.33 %)	2 (2.22 %)	0.153	0.728
Treatment efficiency	90	Significant effect	39	51	–	–
		Valid	42	36	–	–
		Invalid	9	3	–	–
		Total incidence (n/%)	63 (90.0 %)	63 (96.7 %)	4.857	0.038

## 4 Discussion

In the surgery for patients with ankle arthritis, traditional surgical treatment such as incision and repositioning can repair and treat the injury site to restore normal force lines. However, the surgical incision is large, and there are limitations in the treatment of ligament damage and articular surface cartilage. The patient's foot and ankle joint structure cannot be fully restored after receiving traditional surgical treatment, resulting in complications and sequelae that can easily occur during subsequent treatment. The introduction of arthroscopic techniques in the surgical treatment of patients with ankle arthritis can reduce the incision, bleeding and complications at the surgical site, which can help patients recover the function of the foot and ankle joint more quickly [9]. The use of arthroscopic techniques can help healthcare professionals obtain more information about the patient's injury site, such as soft tissue damage and free bone fragments in the foot and ankle joint. Under the guidance of arthroscopic techniques, the health care provider can fix the closure site more precisely, remove the free body from the injured area, and remove the inflammatory synovial membrane, etc. The arthroscopic surgery is better than conventional open surgery for patients with foot and ankle fractures in terms of operative time, treatment time, treatment outcome, and incidence of adverse reactions [10]. The researchers also used arthroscopic techniques in foot and ankle fracture deformity and traumatic arthritis with higher treatment results and patient satisfaction. Meanwhile, the prognosis of patients was effectively improved. Exercise is a necessary rehabilitation exercise after surgical treatment, which can reduce joint injuries and its risk in related studies [11]. For patients with advanced ankle arthritis, the total ankle replacement surgery is a standard treatment [12]. Walking exercises have a positive effect on the postoperative recovery of patients with total ankle arthroplasty, and walking speed and stride length impact foot and ankle's function significantly [13]. Exercises that are too intense can easily cause other secondary injuries such as muscle damage, so the intensity and volume of rehab exercises need to be controlled in the actual treatment.

Human exercise is closely related to BMD, and exercise is beneficial to improving the condition of human bones and reducing the risk of people falling. Physical activity has a better intervention and prevention effect for patients with osteoporosis [14]. 12 weeks of aerobic exercise has a positive effect on patients with reduced BMD [15]. Dual-energy x-ray absorptiometry is commonly used in the treatment of patients with ankle osteoarthritis. It can be used to measure BMD in the wrist and other areas in patients. Meanwhile, there is a correlation between joint damage, wrist function, and osteoporosis [16]. BMD measurements using dual-energy x-ray absorptiometry in different populations showed that adolescents with active and effective exercise had significantly higher BMD than older, less physically active older people [17]. In the postoperative recovery training of arthritis patients, the patients' BMD is closely related to the recovery of joint function. Health care providers need to pay close attention to the BMD of patients, especially in older patients. This group of patients is prone to diseases such as osteoporosis due to lack of exercise, inadequate calcium supplementation or reduced absorption capacity, resulting in decreased BMD levels after joint surgery. Various biochemical indicators such as inflammatory factors or growth factors have an effect on bone growth in humans in studies related to human BMD. The expression of inflammatory factors is significantly increased during human injury. Meanwhile, the body reduces the discomfort caused by trauma by regulating the expression level of inflammatory factors. In an acute gouty arthritis model, drugs can improve the swelling of ankle tissue in rats by inhibiting the expression of inflammatory factors such as IL-6 [18]. TNF-α antagonists have a delayed loss effect on BMD in patients, thereby reducing the incidence of osteoporosis and improving their quality of life [19]. Plant extracts can alleviate the symptoms of arthritis by inhibiting the expression of inflammatory factors such as IL-6 and TNF-α in a rat ankle arthritis model [20]. PDGF is a chemotactic bone growth factor in humans and is an important influence in promoting wound healing and the formation of connective tissue [21]. PDGF promotes the maturation and differentiation of osteoblasts as well as chondrocytes in degenerating joints. PDGF also promotes the division and proliferation of chondrocytes [22]. VEGF is a human growth factor that promotes the production of neovascularization and improves local oxygen and nutrient supply to the body [23]. The body can promote the recovery of patients by promoting local metabolic circulation, enhancing the excretion of metabolites, inhibiting the expression of inflammatory factors, and promoting the synthesis of growth factors [24]. There are relatively few studies on BMD measurement after ankle replacement surgery. The method used in this study better tested the BMD of patients with ankle arthritis compared with the study by Entezari B et al. [25]. This is of great significance for understanding the patient's rehabilitation process. Therefore, the measurement method used in this study has high clinical significance.

Arthroscopic techniques were introduced in this study for the surgical treatment of patients to study the clinical effectiveness of arthroscopic techniques and the necessity of postoperative rehabilitation training. The patients were also allowed to perform light walking for rehabilitation training after surgery in the hope of improving the outcome of patients. The experiment focused on the changes in BMD in patients before surgery and in the postoperative recovery treatment. Meanwhile, correlations were made to analyze the factors influencing BMD. Mild walking combined with arthroscopic surgery had higher Kofoed and AOFAS scale scores in patients (*P* < 0.05), and lower VAS scale scores in Group B (*P* < 0.05) compared to conventional open ankle arthritis surgery. Inflammatory factor levels decreased and growth factor levels increased in both groups after surgical treatment (*P* < 0.05). Inflammatory factors were lower in Group B during the same period (*P* < 0.05), and growth factors were higher in Group B (*P* < 0.05). The BMD growth in Group B was significantly higher (*P* < 0.05) after 2 months after surgical treatment. There was a positive correlation between PDGF bone growth factor and BMD with an R value of 0.957 and between VEGF growth factor and BMD with an R value of 0.903. There was a negative correlation between patient age and BMD with an R-value of −0.936 and between patient BMI and BMD with an R-value of −0.913. Dizziness and abnormal autonomic reflexes occurred in both groups. Meanwhile, the incidence of adverse effects was not significantly different and occurred in a low proportion between the two groups. The treatment efficiency of thw two groups were above 90 %, but the treatment efficiency of Group B was relatively higher (*P* < 0.05). Light walking combined with arthroscopic surgery had a better therapeutic effect on patients with foot and ankle osteoarthritis and improved the BMD level of patients. The treatment also had a better improvement for inflammatory factors and growth factors that affect BMD. Based on these results, the mild walking combined with arthroscopic surgery proposed in this experiment is suitable for the surgical treatment of patients with foot and ankle osteoarthritis.

The advantage of this paper is that the proposed method can effectively improve the physical health status of trauma patients, reduce inflammatory reactions, improve patient's BMD, and reduce the complications. The experiment proves that mild walking combined with arthroscopic surgery has a good therapeutic effect on patients with ankle arthritis. Although the experiment has achieved certain results, the selected comparative indicators in the study have a certain degree of subjectivity. Therefore, it is necessary to evaluate the therapeutic effect from multiple aspects in subsequent research.

## 5 Conclusion

Based on the above research, the Kofoed and AOFAS scores of mild walking combined with arthroscopic surgery are higher than those of conventional treatment. Meanwhile, the pain visual analogue VAS scores are lower than those of conventional treatment. The postoperative BMD growth in the experimental group was significantly higher than that in Group A. Meanwhile, the treatment effectiveness rate in the experimental group was 96.7 %. These results demonstrate that mild walking combined with arthroscopic surgery has a good therapeutic effect on patients with ankle arthritis. This can also improve the BMD level of patients, making it suitable for surgical treatment of ankle osteoarthritis. This provides new options for the treatment and rehabilitation of patients with ankle arthritis.

## Ethics statement

The studies titled “Effect of Light Walking Combined with Arthroscopic Surgery on Bone **Mineral** Density is Patients with Foot and Ankle Osteoarthritis” involving human participants were reviewed and approved by Jeonju University. The patients/participants provided their written informed consent to participate in this study. The patients whose images are included in my paper are agreed to be published. This study was conducted according to the guidelines of the Declaration of Helsinki.

## Funding

This study was supported by the 10.13039/501100014988Scientific Activities of Selected Returned Overseas Professionals in Shanxi Province (20230047).

## Data availability statement

The datasets generated or analyzed during this study are available from the corresponding author on reasonable request.

## CRediT authorship contribution statement

**Xiaoyan Li:** Writing – original draft, Software, Resources, Methodology, Formal analysis, Conceptualization. **Kangwon You:** Writing – review & editing, Methodology, Data curation.

## Declaration of competing interest

The authors declare that they have no known competing financial interests or personal relationships that could have appeared to influence the work reported in this paper.
